# Experience of Labour and Childbirth in a Sample of Portuguese Women: A Cross-Sectional Study

**DOI:** 10.3390/healthcare12212125

**Published:** 2024-10-24

**Authors:** Márcio Tavares, Pedro Alexandre-Sousa, Andrea Victória, Susana Loureiro, Ana Paula Santos, José Mendes

**Affiliations:** 1 Department of Nursing, Family and Community Health, School of Health, University of the Azores, 9500 Ponta Delgada, Portugal; marcio.fm.tavares@uac.pt (M.T.); 199803@uac.pt (A.V.); ana.ps.santos@uac.pt (A.P.S.); 2 Center for Innovative Care and Health Technology (ciTechcare), Polytechnic of Leiria, 2411 Leiria, Portugal; pesousa@arscentro.min-saude.pt; 3 Hospital do Santo Espírito Santo da Ilha Terceira, 9700 Angra do Heroísmo, Portugal; sl810829@azores.gov.pt; 4 Insight: Piaget Research Center for Ecological Human Development, 2805 Almada, Portugal

**Keywords:** labour, obstetric, parturition, pregnant woman, life experience

## Abstract

Background/Objectives: Childbirth is a profoundly personal experience that often does not align with expectations. The World Health Organization has established guidelines for best practises; in this sense, it is crucial to understand the childbirth experiences of Portuguese women in comparison with these guidelines. Methods: A quantitative, descriptive, correlational, and cross-sectional study was conducted to achieve this. In total, 615 women completed a sociodemographic questionnaire and the Labour and Childbirth Experience questionnaire, which comprised 39 statements based on the WHO’s recommendations. Additionally, the study utilized the Life Satisfaction Scale and gathered insights into participants’ overall perception of care during this phase. Results: The results were categorized as follows: (1) practises influencing the labour experience; (2) practises influencing the experience of vaginal birth; (3) practises affecting the experience of caesarean birth; and (4) emotional experience during labour and birth. Conclusions: Notably, the study found that practises discouraged by the WHO are still prevalent, potentially enabling obstetric violence. However, a robust and statistically significant correlation was observed between the childbirth experience and the overall perception of care.

## 1. Introduction

Pregnancy is a phase in the life cycle that brings critical transitions in family life. It is typically a time of numerous dreams and expectations, which are emotions that play a vital role in a woman’s childbirth experience [1]. However, the experience of childbirth rarely aligns with a woman’s expectations. This disparity between expectations and reality impacts a woman’s satisfaction with childbirth and the health professionals providing care during childbirth. [2]. Furthermore, the World Health Organization (WHO) warns that the increasing medicalization of childbirth affects women’s experience and satisfaction during this process [3]. In this sense, health professionals must provide quality care, prevent obstetric mistreatment, and promote literacy on the subject [4]. The following have been identified as acts of mistreatment by the authors: negligence, verbal abuse, violation of privacy, and unnecessary procedures that go against good medical practise or are not consented to by the woman. In recent years, the rising number of reports on online platforms such as Facebook and Instagram about women experiencing mistreatment during childbirth has brought public attention to this issue, making it an area of interest for the WHO and the United Nations [5].

The study of Lundh et al. [6] examines the emotional and psychological impacts of unexpected labour induction on women’s experiences, highlighting feelings of loss of control and the need for better communication from healthcare providers. Viirman et al. [7], in their work “Negative childbirth experience in relation to mode of birth and events during labour: A mixed methods study”, found that there is a link between negative birth experiences to specific labour events and modes of delivery, emphasizing the importance of informed consent and respectful care.

The WHO defines childbirth as a natural physiological event that typically does not necessitate interventions. It emphasizes the need for the careful consideration of practises during labour and delivery, guided by scientific evidence and compassionate care, to ensure the safety and well-being of both the mother and the baby. As a result of thorough research, the WHO has developed 56 evidence-based recommendations about childbirth practises [3]. These recommendations arise from the need to ensure safety and well-being, but also to reduce obstetric violence.

Despite initial criticisms of excessive medicalization dating back to the 1960s and 1970s, it was only in 2014 that the World Health Organization formally recognized obstetric violence in its declaration on the prevention and elimination of abuse and mistreatment during childbirth in healthcare facilities. Latin America was at the forefront of regulating obstetric violence, with Argentina (2004) and Venezuela (2007) taking early action.

In Europe, the issue gained traction with the Council of Europe’s 2019 resolution 2306/2019, which urged member states to combat obstetric violence. Countries like Spain, Italy, and France have since made legislative efforts in this area. In Portugal, with 1 in 3 women reporting obstetric violence (https://shre.ink/g3br, accessed on 18 October 2024), law 110/2019 established patient rights in sexual and reproductive healthcare, including specific protections for women as mothers or potential mothers. However, the term “obstetric violence” was only explicitly used in Portugal’s resolution 181/2021, which recommends government action for its eradication.

Although Portuguese women already had legal protection, the absence of the explicit recognition of obstetric violence has kept the issue relatively invisible in legal and judicial contexts. Efforts to introduce penalization and improve training for healthcare professionals continue, as highlighted by recent legislative initiatives from political parties.

Therefore, it is crucial to understand women’s experiences during labour and childbirth in Portugal and compare them with identified best practises.

## 2. Materials and Methods

### 2.1. Design

A quantitative, descriptive, correlational, and cross-sectional study was conducted in mainland Portugal and its autonomous regions (Madeira and the Azores).

### 2.2. Participants

The study involved 615 women who met the following inclusion criteria: age 18 years or older and had had a pregnancy less than five years ago without interoccurrence. The average age was 34.02 years (standard deviation (SD) = 5.01), ranging from 19 to 48 years. Regarding education, 65.7% received higher education, 29.3% received secondary education, and 5% received primary education. Regarding marital status, 88.8% were in a relationship and lived with their partner (50.9% married, 37.9% in a de facto union), while 11.2% were not in a relationship (9.8% single, 1.4% divorced).

All the participants (99.5%) had pregnancy monitoring consultations, with 21.3% seeing their family doctor, 57.4% seeing a private doctor, 1.6% seeing a midwife, and 19.7% attending in-hospital consultations. It is important to note that 59.8% of the women had participated in childbirth preparation programmes.

Regarding the type of birth, 39.7% had a typical vaginal birth, 3.9% had a planned caesarean section, 33.8% had an emergency caesarean section, 18.7% had a vacuum extraction birth, and 3.9% had a forceps birth. The average age of the women at the time of delivery was 32.19 years, and 62.3% were first-time mothers.

Regarding residence, 53.2% lived in mainland Portugal, 40.3% in the Azores, and 6.5% in Madeira. Portugal’s healthcare system is organized across three distinct regions—the Autonomous Region of the Azores, the Autonomous Region of Madeira, and the mainland. Each of these regions has its own healthcare system, managed by the respective regional government in the case of the Azores and Madeira, and by the national government for the mainland. Although they share certain principles, such as the universal nature of healthcare and funding through taxes, the organization, management, and implementation of healthcare services vary significantly between these regions.

This structure reflects Portugal’s constitutional commitment to regional autonomy, allowing the Azores and Madeira to tailor their healthcare systems to meet their unique challenges while still being integrated into the broader national health framework.

### 2.3. Data Collection and Measures

#### 2.3.1. Sociodemographic Questionnaire

A sociodemographic questionnaire was created to gather information on whether the participants had been mothers for less than five years, the type of birth, their age when they became mothers, their educational qualifications, marital status, place of residence, number of children, preparation for childbirth, and whether they had received pregnancy monitoring.

#### 2.3.2. Labour and Childbirth Experience Questionnaire (LCEQ)

The LCEQ comprises 39 statements that have undergone multidisciplinary reflection, guided by the WHO’s recommendations for a positive birth experience [3].

The statements were organized and presented using a Likert scale. Each item allows for a range of responses between 1 and 4. A reaction of 1 corresponds to maximum disagreement, while a response of 4 corresponds to maximum agreement. Options 1 and 2 indicate negative experiences, while options 3 and 4 indicate positive experiences, except for items with inverted scores. The following items were inverted: 1, 5, 6, 8, 10, 11, 12, 15, 16, 17, 19, 22, 23, 27, 29, 32, 34, 35, 37, 38, 39, and 41. Items 39 to 42 are exclusive to women who have undergone a caesarean section.

#### 2.3.3. Satisfaction with Life Scale

The Satisfaction with Life Scale (SWLS) [8] was used to measure life satisfaction. It consists of five items rated on a Likert scale, with 1 = strongly disagree, 2 = somewhat disagree, 3 = neither agree nor disagree, 4 = somewhat agree, and 5 = strongly agree. Scores can range from 5 to 25, with higher scores indicating greater life satisfaction. The reliability coefficient for this study was 0.88.

#### 2.3.4. Women’s Perception of Care

The question “On a scale of 1 to 10, how well did you feel cared for?” was asked to gauge the overall perception of care provided to women during labour. In this context, a rating of 1 signified feeling inadequately cared for, whereas a rating of 10 denoted feeling exceptionally well cared for.

#### 2.3.5. Procedures

Participants were recruited through two channels. Initially, we disseminated calls for study participation via social media (e.g., Facebook and Instagram) and shared the study link. Additionally, we prepared informative leaflets detailing the study’s objectives and participant criteria that were distributed in strategic locations such as kindergartens, health centres, and doctors’ offices. Notably, we eschewed the collection of any identifying information from participants. A total of 982 responses were obtained, of which 367 were excluded for not meeting the inclusion criteria. The study protocol was uploaded onto the Survey 123 platform and promoted across social networks, including Facebook and Instagram.

#### 2.3.6. Statistical Analysis

Following the collection of questionnaires, the data were entered and analyzed using IBM^®^ SPSS Statistics software, version 28.0.0 for macOS. Subsequently, a Kolmogorov–Smirnov test was conducted, revealing that the data did not meet the assumptions of normality required for parametric tests at a significance level of <0.001. Descriptive statistics and dispersion measures were employed, along with Spearman’s rank correlation coefficient, to examine correlations. The Kruskal–Wallis and Wilcoxon–Mann–Whitney tests were utilized to assess statistically significant differences.

## 3. Results and Discussion

Upon analysis, it was found that there were correlations between the items in the LCEQ (correlation coefficient of 0.77) and the Satisfaction with Life Scale (correlation coefficient of 0.88). Cronbach’s alpha values for the questionnaire on the perception of labour and the SLWS indicate acceptable internal consistency for the scales used [9]. The findings from the survey on women’s labour experience reflect the WHO’s guidelines for a positive birth, as seen in a sample of Portuguese women [3].

**Figure 1:**
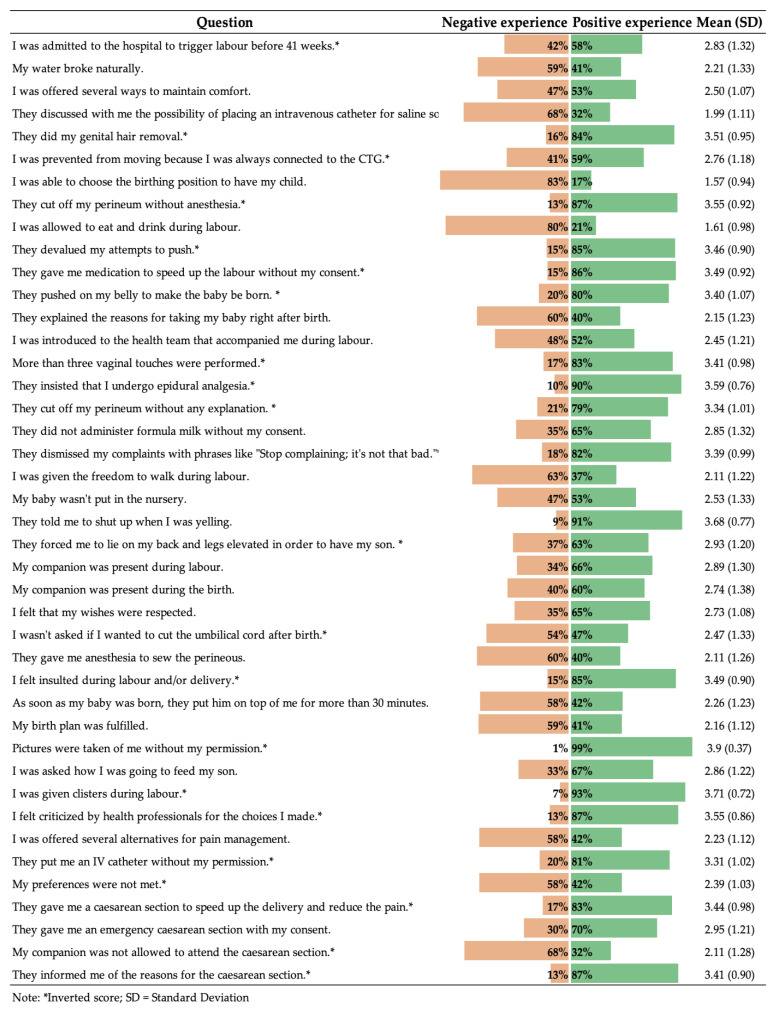
Results of Labour and Childbirth Experience Questionnaire (LCEQ).

Figure 1 displays the results of the LCEQ, indicating whether the participants perceive it as a positive or negative experience.

In response to inquiries about their overall perception of care, participants provided a range of scores from 1 to 10, with 1 indicating poor care and 10 indicating excellent care. On average, the participants rated their care at 7.32, with an SD of 2.20. Notably, 10.7% of the women reported experiencing less positive care, with scores between 1 and 3. However, the majority (55.3%) perceived the care as positive, scoring eight or higher. As shown in Table 1, the analysis revealed a weak but statistically significant correlation between the experience of labour and life satisfaction (r = 0.21, *p* < 0.001), as well as a strong and statistically significant correlation between labour experience and overall perception of care (r = 0.62, *p* < 0.001). The labour experience displayed weak correlations with sociodemographic variables, except for the type of delivery, which exhibited a moderate and statistically significant negative correlation (r = −0.32, *p* < 0.001). Furthermore, the Kruskal–Wallis test indicated significant differences between the type of birth and labour experience (H(2) = 53.1; *p* < 0.001; N = 615). Upon conducting multiple comparisons (Kruskal–Wallis), it was found that typical vaginal birth exhibited a substantially different distribution of labour experience compared to caesarean birth (*p* < 0.001) and instrumental birth (*p* < 0.001).

### 3.1. Analyzing the Variables

The prevalence of mistreatment during childbirth varies significantly between countries. A study conducted in four African and Asian countries identified obstetric abuse in over 40% of the observed births and over 35% of the surveyed women [10]. In contrast, a study conducted in six European countries estimated that, on average, one in five women experience abusive healthcare during pregnancy [11]. Given the divergence across countries, the WHO recommends researching non-compliance with proper obstetric practises in various contexts to better understand its implications for women’s experiences and decision-making [12]. To enhance data management, the variables have been categorized into the following four groups: (i) factors impacting the labour experience; (ii) factors influencing the experience of vaginal birth; (iii) factors affecting the experience of caesarean section; and (iv) emotional experiences during labour and birth.

Furthermore, for interpretation, the 4-point Likert scale options were dichotomized into two categories—agree (3–4) and disagree (1–2).

#### 3.1.1. Practises That Influence the Experience of Labour

**Figure 2:**
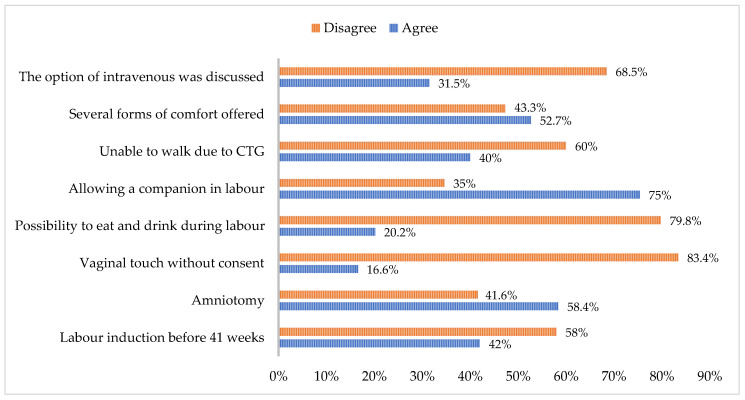
Practises that influence the experience of labour.

Figure 2 displays the percentages related to practises affecting the labour experience. This includes the following grouped items: induction of labour before 41 weeks, amniotomy, vaginal examinations without consent, permission to eat and drink during labour, presence of a companion during labour, inability to walk due to continuous monitoring, various forms of comfort provided, and discussion of the option for intravenous intervention.

In the context of inducing labour before 41 weeks of gestation, Figure 2 illustrates that 42% of the women surveyed acknowledged this practise. Among the participants, 42% disclosed that they were hospitalized before 41 weeks for labour induction, with the highest consensus indicating gestational ages between 41 and 42 weeks [13]. When advising mothers about the potential need for labour induction, it is essential to consider their perceptions of childbirth and to collectively reach a decision. This approach is crucial for preventing the mother from feeling pressured into undergoing labour induction [14]. The induction of labour is a medical procedure aimed at initiating uterine contractions when the mother is not in active labour. Refraining from this intervention before 39 weeks of gestation is generally recommended and should only be pursued when it is deemed unsafe to extend the pregnancy further [15,16]. It is essential to uphold the pregnancy and expectant behaviour when a lower induction benefit is anticipated. Labour induction between 41 + 2 and 41 + 3 weeks has been associated with a reduction in perinatal mortality. Furthermore, a decrease in stillbirths across all gestational age groups from 2 days to 41 weeks was demonstrated in comparison to expectant behaviour. However, it is noteworthy that the induction of labour has been correlated with a higher incidence of low Apgar scores at 5 min and admissions to neonatal units within 24 h of birth, coinciding with elevated rates of caesarean section. When advising women at 41 weeks of gestation, these findings should be deliberated upon regarding the choice between the induction of labour and expectant behaviour [15,16]. There is a growing acknowledgment of the necessity to initiate labour before 41 weeks of gestation due to a significant association between a high incidence of inductions and caesarean sections. Through the Wilcoxon–Mann–Whitney test, statistically significant differences were found (U = 54.034; *p* < 0.001; N = 615).

Amniotomy is a procedure often utilized to expedite labour progression. However, according to the WHO’s recommendation [3], it is suggested to consider avoiding this practise due to the lack of discernible benefits in labour outcomes and the potential for an elevated incidence of birth-related adverse events. Nevertheless, it is noteworthy that this procedure is prevalent in Portuguese maternity wards, as indicated by 58.4% of women who have reported undergoing amniotomy.

Vaginal touching without permission still happens, with 16.6% of women reporting it. The perpetuation of this violation of privacy is unacceptable and must be addressed. The WHO [3] says that vaginal touch is only recommended during the active phase of the first stage of labour, with at least four hours between each check. The first stage of labour usually lasts between 2 and 7 h. So, only one or two touches should happen, and only with permission. The WHO also emphasizes the need for respectful and private care for a positive birth experience [17].

An important revelation from this study was that 79.8% of the women could not consume food or drinks during labour. According to the WHO [3], women with low-risk pregnancies should be permitted to ingest fluids and food during labour. This practise has been found not to pose an increased risk of adverse outcomes for the woman or the fetus/baby. On the contrary, it has been associated with heightened satisfaction and cooperation among women during labour [18].

Psychological and emotional well-being are crucial factors for improved health outcomes. A supportive individual during labour is considered essential for women’s well-being and is recommended in all the available evidence [3,19,20,21]. However, as shown in Figure 2, 35% of women did not have the opportunity to be accompanied during their labour, and 60% reported feeling constrained in their movements due to constant monitoring (cardiotocography). Continuous monitoring during labour is not indicated. Cardiotocography (CTG) is only recommended in high-risk cases, as per the WHO guidelines. It can cause distress and limit movement, and it is associated with higher rates of caesarean sections and challenging vaginal deliveries [3,22].

Upon inquiry, 47.3% of the participants affirmed not receiving various forms of comfort from the professionals. According to the WHO [3], relaxation techniques, including progressive muscle relaxation, breathing exercises, music therapy, mindfulness, and other modalities, are recommended for pain management during labour, considering the woman’s preferences [20].

A total of 31.5% of participants discussed the possibility of IV access, while 68.5% did not. The evidence shows no benefit in using IV fluid infusion to promote labour progression [3]. Opting out of IV catheterization in uncomplicated cases is reasonable if urgent access is available [21]. The practical effect of placing an IV catheter is questioned without discussing the option of doing so.

#### 3.1.2. Practises That Influence the Experience of Vaginal Birth

**Figure 3:**
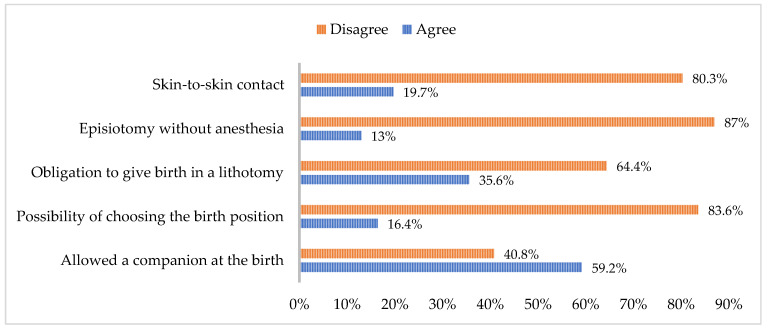
Practises that influence the experience of vaginal birth.

The following variables were categorized together as factors influencing the childbirth experience: the opportunity to have a support person present during birth, the ability to select a birthing position, the requirement to assume a gynecological position during delivery, the administration of episiotomy without anesthesia, and the facilitation of skin-to-skin contact. Figure 3 presents these grouped variables, and the percentage of concurrence or discordance expressed by participants in response to the survey questions.

According to recent research, 40.80 percent of the surveyed women did not have the option of having a companion during childbirth. The WHO [3] recognizes and upholds the right of all women to be accompanied by a chosen relative or person during childbirth, acknowledging the positive impact this can have on the well-being of the mother and the newborn [23]. Additionally, the study found that 83.6 percent of participants were not given the option to choose their birthing position, with 64.4 percent reporting being forced to give birth in the lithotomy position. Extensive evidence exists on the benefits of varied labour positions, as indicated by multiple studies [24,25,26,27].

The WHO recommends encouraging women to adopt upright positions during labour [3] as a practise for low-risk pregnancies. Refusing to allow women to choose their birthing position or not informing them of this option is considered a form of obstetric violence and should be avoided [25,27]. Meijer et al. [27] define this as a component of obstetric violence and found that 37.2% of participants in their study were not given the choice of position. Vertical positions, which do not pose different risks compared to horizontal positions, have been linked to favourable outcomes such as shorter labour, better uterine involution, improved adaptation to life outside the uterus [28], reduced pressure on the baby’s neck during delivery [26], and a lower incidence of perineal injuries [29].

When episiotomies were performed, 13% of women reported that the procedure was carried out without any form of anesthesia. The WHO [3] proposes a target of zero for the percentage of episiotomies performed without anesthesia and recommends avoiding this practise as a routine by investing in practises that promote perineal protection.

Skin-to-skin contact is a recommended practise for stimulating the initiation of breastfeeding and preventing postpartum hemorrhages by stimulating the production of oxytocin [30,31]. The failure to promote this practise can lead to hemorrhages, a low adherence to breastfeeding, and a reduced stimulation of emotions and memory, which, in turn, inhibits oxytocin secretion and weakens attachment. This can have long-term effects on the development and stabilization of vital parameters. A study showed that 80.3% of women did not have skin-to-skin contact in the first hour after giving birth.

#### 3.1.3. Practises That Influence the Experience of Caesarean Section

**Figure 4:**
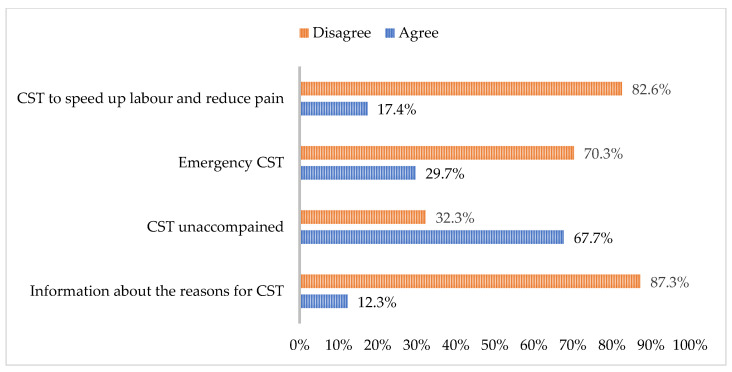
Practises that influence the experience of caesarean section.

The data presented in Figure 4 indicate that 87.3% of the women surveyed reported being informed about the clinical reasons for doctors recommending a caesarean section. However, 12.7% of respondents acknowledged not receiving this information. The prevalent perceived justifications for caesarean sections, as reported by the women, include emergencies (70.3%), and the procedure is utilized to expedite birth and alleviate pain (82.6%). Despite this prevalent perception, it is essential to note that the National Institute for Health and Care Excellence (NICE) recommends that women be empowered with information and be involved in decision-making. Notably, the NICE’s 2021 guide, updated in 2024, does not endorse expediting birth and pain management as valid justifications for a caesarean section [32].

Furthermore, although the right to have a companion during a caesarean section is legally established in Portugal, 67.7% of women indicated that they did not have this option, and some were even declined. Women undergoing caesarean sections benefit from the continuous support of their chosen companions, feeling empowered to make decisions and comforted by the ongoing assistance [33].

#### 3.1.4. Emotional Experience During Labour and Birth

**Figure 5:**
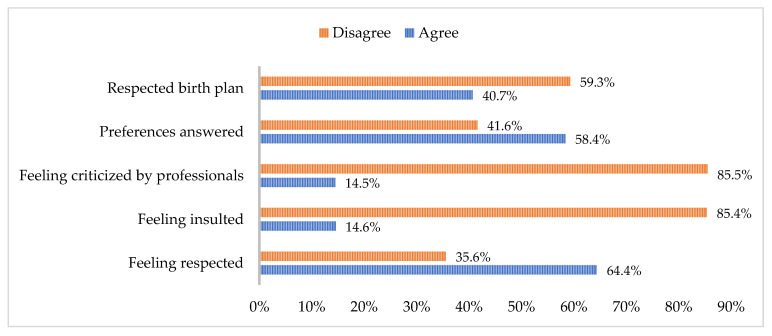
Emotional experience during labour and birth.

Several variables were combined regarding the emotional experience during labour and birth, including feeling respected, feeling insulted, feeling criticized by professionals, having preferences met, and having the birth plan respected. Figure 5 illustrates the grouping of these variables along with the percentage of agreement or disagreement among the participants in response to the questions they were asked.

The participants’ responses indicate that there is still progress to be made in this area. While 35.6% of the participants reported feeling generally respected, a significant 85.4% stated that they had felt insulted at some point, and 85.5% felt criticized by health professionals. It is recommended that respectful maternity care be provided to all women in a manner that preserves their dignity, privacy, and confidentiality. This care should prevent harm and mistreatment and allow for informed choices and continuous support during labour and childbirth [3].

The need for emotional support, including being present during labour, clear communication, and respectful care, is often overlooked and not considered a priority in many settings [34]. This leads to disrespectful and undignified treatment in healthcare facilities worldwide, particularly affecting vulnerable populations. This violates their human rights and creates a significant barrier to accessing essential childbirth services [3].

Regarding the participants’ responses on whether their preferences were honoured and if the birth plan was respected, the findings indicate that 41.6% and 59.3% of the women, respectively, reported that their wishes were not fulfilled. There is a lack of widespread respect for women’s preferences. Providing respectful care aligns with a human rights-based approach, which has the potential to enhance women’s experience during labour and childbirth and address health disparities. Additionally, it contributes to reducing maternal morbidity and mortality [3].

Effective communication and engagement among healthcare providers, health service managers, women, and women’s rights movements are crucial to guarantee that care aligns with women’s needs and preferences across all contexts and settings [3]. Birth plans must also be tailored to each woman’s needs and preferences [3].

The participants expressed positive satisfaction with life, with values averaging higher than the midpoint of 19.11 (SD = 4.10). The current study supports the link between pregnancy [30] and motherhood [31] and positive satisfaction in women’s lives.

The results indicated that women perceived caesarean delivery less positively. These findings align with a study by Burcher et al. [35] that revealed that factors such as poor communication, fear of the operating room, distrust in health professionals, and loss of control impact the dissatisfaction of women giving birth. It is worth noting that cultural influences may also shape these results related to care provision [36].

Evaluating various aspects of care during labour and delivery revealed a more diverse and sometimes negative perception compared to the overall evaluation of women’s experiences [37]. This is evident in our study, with negative experiences reported in specific areas and a positive overall perception of the care received. These findings are consistent with other studies conducted in Portugal [37,38,39] that also reported high satisfaction among women with the care received during labour and delivery. Notably, no study in Portugal has indicated low satisfaction among women with childbirth.

Despite the recent focus on assessing women’s satisfaction with peripartum care, only a few validated instruments designed for specific populations [37] exist. Thus, instruments to facilitate this evaluation need to be developed and validated.

The results of this study on childbirth experiences and obstetric violence in Portugal may vary across different regions or healthcare settings due to several factors. Because Portugal’s healthcare system is decentralized, each region faces unique challenges, such as access to specialized care, resource distribution, and staff training. The more remote areas may experience greater disparities in healthcare practises compared to urban centres in the mainland, potentially exacerbating issues of obstetric violence due to limited resources and specialized training. The study’s findings on obstetric violence and childbirth experiences highlight gaps in adherence to WHO guidelines across Portugal, but the impact can vary significantly depending on regional healthcare disparities, as well as socioeconomic and cultural factors. These variations underscore the need for systemic changes that ensure equitable access to respectful and evidence-based maternity care across all regions and healthcare settings in Portugal.

In this sense, this study highlights several areas within Portugal’s healthcare system that impact women’s childbirth experiences, with some practises diverging from the WHO guidelines. These findings offer significant insights into how healthcare systems might address these issues and improve maternal care.

One of the central themes in the data is the prevalence of obstetric violence, including forced positions during childbirth, non-consensual vaginal examinations, and episiotomies performed without anesthesia. The WHO explicitly discourages such practises, advocating for a woman’s right to informed consent and respecting her preferences. Obstetric violence not only violates human rights, but also undermines trust in healthcare systems and negatively impacts the emotional and psychological well-being of women.

To address these issues, healthcare systems should implement stricter protocols for obtaining informed consent, ensuring that all procedures are clearly explained, and the patient’s consent is obtained in advance. Also, healthcare professionals should be trained to recognize and prevent obstetric violence, emphasizing respect for patient autonomy and rights. Healthcare systems must create and enforce accountability mechanisms, establishing systems where women can report violations without fear of reprisal, ensuring that healthcare providers adhere to guidelines promoting dignified and respectful care, improving communication and decision-making where women must be actively involved in discussions about their birthing options, with clear information on risks, benefits, and alternatives. Re-evaluating policies to ensure that companions are allowed and encouraged during both vaginal and caesarean births is a measure that must be taken. Physical comfort and mobility, revising labour protocols to align with WHO guidelines, adopting intermittent monitoring for low-risk pregnancies promoting mobility, and reducing caesarean rates, which can improve birth experiences and outcomes, are some areas that must be subjected to intervention. Alternative comfort measures, such as relaxation techniques and non-pharmacological pain management options, help women feel more empowered and satisfied during labour; respect for birth preferences and emotional support improve the integration of birth plans into care, fostering a culture of emotional support and communication, promoting vaginal birth as the first choice; and empowering women through better education on caesarean sections, helping them understand when they are necessary and dispelling myths around their convenience or safety, are some fields that demand attention in Portugal.

There are some important limitations of the present study that should be discussed. Firstly, the sample is not representative of the entire Portuguese population. Secondly, the influence of social networks and multiple cases of obstetric violence on media was not explored, as well as its influence on women’s answers. Third, no distinction was made between public, private, and academic hospitals. Future studies should focus on the perception of this phenomenon.

## 4. Conclusions

The experience of childbirth is an intensely personal one and can be shaped by a variety of factors. The relationship formed with healthcare professionals plays a significant role among these factors. Given the numerous reports in the media regarding instances of obstetric violence, it is essential to gain a clear understanding of the actual prevalence of such situations. Consequently, we undertook this study to comprehend women’s experiences during labour and childbirth in Portugal and to compare them with recognized best practises. Our analysis revealed that the recommendations of the WHO are not yet widely adopted by healthcare professionals, leading to the use of practises identified as obstetric violence. Additionally, professionals are implementing practises that impact the experience of both vaginal and caesarean section births. A notable percentage of women reported feeling insulted, criticized, or disrespected by these professionals. Despite this, we observed a strong and statistically significant correlation between the childbirth experience and the overall perception of care. This study has the potential to prompt professionals to contemplate their practises and recognize the necessity of implementing new evidence-based practises. Furthermore, it underscores the importance of empowering women to advocate for their right to receive care from professionals trained in evidence-based practises.

This study makes an important scientific contribution by highlighting gaps in the application of evidence-based childbirth practises in Portugal and how these practises affect women’s childbirth experiences. Specifically, it sheds light on the prevalence of obstetric practises that do not align with WHO guidelines and how these practises negatively impact births. It also examines emotional factors such as feelings of disrespect, insult, and criticism during labour, emphasizing the need for improved communication and respectful care to ensure a positive childbirth experience. This aligns with the growing recognition of the importance of emotional well-being and respectful maternity care in improving maternal health outcomes. Moreover, the research offers data on underexplored areas, like the high prevalence of obstetric violence in Portugal, and how this impacts women’s perceptions of care. This is crucial for improving healthcare policies and practises to reduce maternal morbidity and mortality. It adds valuable empirical data that calls for healthcare professionals to adopt WHO guidelines and focus on women’s preferences and rights during childbirth, pushing for systemic changes in maternal healthcare.

## Figures and Tables

**Table 1:** Correlations between the Questionnaire on the Experience of Labour and Childbirth (QETPP), life satisfaction, and sociodemographic variables (N = 615).

	1	2	3	4	5	6	7	8	9	10	11
1. QETPP	__	0.21 ***	−0.32 ***	0.10 **	0.09 *	0.06	−0.14 ***	0.05	−0.12 **	−0.01	0.62 ***
2. SLWS		__	−0.01	0.17 ***	0.18 ***	−0.02 ***	0.09 *	0.11 *	0.04 *	0.04	0.34 ***
3. Type of labour			__	−0.01	0.01	0.01	−0.07	−0.17 ***	0.01	0.01	−0.11 **
4. Age when becoming a mother				__	0.32 ***	0.01	0.03	0.31 ***	−0.06	−0.02	0.15 ***
5. Educational qualifications					__	0.03	−0.02	−0.13 **	−0.30 ***	−0.04	0.04
6. Marital status						__	−0.13 **	−0.8	−0.07	0.01	0.05
7. Place of residence							__	0.13 **	0.18 ***	−0.02	−0.06
8. Number of children								__	0.21 ***	−0.01	0.11 **
9. Labour preparation									__	−0.01	−0.02
10. Pregnancy monitoring										__	0.03
11. Overall satisfaction											__

Note: Labour and Childbirth Experience Questionnaire (LCEQ); SLWS = Satisfaction with Life Scale; * *p* < 0.05; ** *p* < 0.01; *** *p* < 0.001.

## Data Availability

The data presented in this study are not available due to privacy restrictions.
